# Do hypoallergenic cats exist? -- Determination of major cat allergen Fel d 1 production in normal and hypoallergenic cat breeds

**DOI:** 10.1186/2045-7022-4-S2-P11

**Published:** 2014-03-17

**Authors:** Julia Satorina, Krisztina Szalai, Anna Willensdorfer, Nadine Mothes-Luksch, Anna Lukschal, Erika Jensen-Jarolim

**Affiliations:** 1 Institute of Pathophysiology and Allergy Research, Waehringer Guertel 18-20, Vienna, 1090, Austria; 2 University of Veterinary Medicine Vienna, Messerli Research Institute, Vienna, Austria; 3 Medical University of Vienna, Institute of Pathophysiology and Allergy Research, Vienna, Austria; 4 Medical University of Vienna and Veterinary Medicine, Inst. of Pathophysi. and Allergy R.,Messerli Inst., Vienna, Austria

## Background and aims

Due to the increasing prevalence of cat allergy the demand for so-called hypoallergenic cats and the number of respective breeders is rising. The aim of our study was to examine the hypoallergenicity of these cats, taking the major allergen Fel d 1 as a marker molecule. This molecule is an important asthma inducer and primarily produced in the sebaceous, salivary and anal glands and is distributed on the fur by licking.

## Methods

We collected samples from 6 normal and 8 hypoallergenic cats by stroking with absorbent cotton over the face, chest and saliva. Allergens were extracted and Fel d 1 levels analyzed in each sample using a commercial ELISA-Kit. The allergen content of the samples was analyzed on SDS-PAGE. IgE binding activity was tested by immunoblot under reducing and non-reducing conditions using sera of cat allergic patients and monoclonal anti-Fel d 1 antibody.

## Results

Total Fel d 1 levels were reduced in sampels from the face and even more in those from the chest of hypoallergenic cats. IgE binding of human patients sera with cat samples showed that only under non-reducing conditions signals were detectable at 18 and 35kDa. Additionally, samples of normal cats showed stronger IgE binding than hypoallergenic cat samples. The monoclonal anti-Fel d 11 antibody showed stronger binding and detected two bands at 18 and 35kDa in normal cats. In contrast less intensive and only a single Fel d 11 band was detected at 18kDa in the samples from hypoallergenic cats.

## Conclusion

Based on our data we conclude that hypoallergenic cats secrete and distribute less Fel d 1 as compared to normal cats to their fur coat. We propose that indeed, hypoallergenic cat breeds pose an attractive alternative for atopic or cat allergic patients.

Supported by the Austrian Science Fund grant F4606-B19.

